# Prognostic impact of in-hospital hemoglobin decline in non-overt bleeding ICU patients with acute myocardial infarction

**DOI:** 10.1186/s12872-023-03251-6

**Published:** 2023-05-03

**Authors:** Pengfei Li, Meixiang Chen, Yuekang Huang, Ruixin Wang, JiaNing Chi, Jiaman Hu, Jianyu Huang, Ningxia Wu, Hua Cai, Hui Yuan, Min Li, Lin Xu

**Affiliations:** 1 The First School of Clinical Medicine, Southern Medical University, Guangzhou, China; 2 Department of Geriatric Cardiology, General Hospital of Southern Theater Command, Guangzhou, China; 3 Branch of National Clinical Research Center for Geriatric Diseases, Chinese PLA General Hospital, Guangzhou, China

**Keywords:** Hemoglobin, Acute myocardial infarction, Non-overt bleeding, Mortality, ICU

## Abstract

**Background:**

The prognostic value of in-hospital hemoglobin drop in non-overt bleeding patients with acute myocardial infarction (AMI) admitted to the intensive care unit (ICU) remains insufficiently investigated.

**Methods:**

A retrospective analysis was performed based on the Medical Information Mart for Intensive Care (MIMIC)-IV database. 2,334 ICU-admitted non-overt bleeders diagnosed with AMI were included. In-hospital hemoglobin values (baseline value on admission and nadir value during hospitalization) were available. Hemoglobin drop was defined as a positive difference between admission and in-hospital nadir hemoglobin. The primary endpoint was 180-day all-cause mortality. The time-dependent Cox proportional hazard models were structured to analyze the connection between hemoglobin drop and mortality.

**Results:**

2,063 patients (88.39%) experienced hemoglobin drop during hospitalization. We categorized patients based on the degree of hemoglobin drop: no hemoglobin drop (n = 271), minimal hemoglobin drop (< 3 g/dl; n = 1661), minor hemoglobin drop (≥ 3 g/dl & < 5 g/dl, n = 284) and major hemoglobin drop (≥ 5 g/dl; n = 118). Minor (adjusted hazard ratio [HR] = 12.68; 95% confidence interval [CI]: 5.13–31.33; *P* < 0.001) and major (adjusted HR = 13.87; 95% CI: 4.50-42.76; *P* < 0.001) hemoglobin drops were independently associated with increased 180-day mortality. After adjusting the baseline hemoglobin level, a robust nonlinear relationship was observed in the association between hemoglobin drop and 180-day mortality, with 1.34 g/dl as the lowest value (HR = 1.04; 95% CI: 1.00-1.08).

**Conclusion:**

In non-overt bleeding ICU-admitted patients with AMI, in-hospital hemoglobin drop is independently associated with higher 180-day all-cause mortality.

**Supplementary Information:**

The online version contains supplementary material available at 10.1186/s12872-023-03251-6.

## Introduction

Acute myocardial infarction (AMI) is a critical form of coronary heart disease caused by atherosclerosis-related plaque rupture and thrombosis. In accordance with the Global Burden of Disease Study 2017, the global incidence of AMI was 106.37/100,000, and the patients gradually tended to be younger, making it the leading cause of death and disability among patients with coronary heart disease [1–3]. The introduction of novel antithrombotic medications and prompt revascularization strategies has significantly improved the prognosis of AMI patients in recent decades [4]. However, the widespread use of dual anti-platelet therapy exposes patients to higher bleeding risk [5, 6].

Bleeding events are strongly associated with high mortality in acute coronary syndromes (ACS) and those undergoing percutaneous coronary intervention (PCI) [7–9]. However, contemporary definitions of bleeding endpoints often exclude occult bleeding events, making it crucial to quantify bleeding risk in non-overt bleeding AMI patients and evaluate their impact on prognosis [10]. In-hospital hemoglobin decline is a significant component of bleeding assessment scales in AMI [11, 12]. A reduction of 3 to 5 g/dl hemoglobin was used to categorize bleeding severity in clinical trials and registries [13–15]. Some definitions already incorporate hemoglobin drop without overt bleeding [16–18]. A recent study [19] revealed that in-hospital hemoglobin reduction ≥ 3 g/dl was the independent risk factor for 1-year all-cause mortality in ACS patients treated invasively, even without overt bleeding events. AMI patients admitted to the intensive care unit (ICU) are often characterized by poor general condition and few opportunities for invasive intervention. Hemoglobin decline may occur without any sign of blood loss during their ICU stay.

The association between in-hospital hemoglobin drop and all-cause mortality in non-overt bleeding ICU patients with AMI remains unknown. This study aims to investigate the relationship using the Multiparameter Intelligent Monitoring in Intensive Care (MIMIC-IV) database.

## Method

### Data source

We conducted a large sample retrospective analysis using the MIMIC-IV database (version 1.0) [20]. This database is developed by the computational physiology laboratory of the Massachusetts Institute of Technology (MIT, Cambridge, MA, USA) with approval from the Institutional Review Boards (IRB) of MIT and Beth Israel Deaconess Medical Center (BIDMC), contains demographic data, vital signs, laboratory results, and prescriptions collected between 2008 and 2019 in the ICU or emergency department of BIDMC [21]. Patient identifications were removed to comply with the Health Insurance Portability and Accountability Act’s (HIPAA) Safe Harbor provision. One author, Pengfei Li, completed the online course on Data or Specimens Only Research and gained access to the database for data extraction after passing the Collaborative Institutional Training Initiative (CITI) exam (Record ID: 45,823,456).

### Study population and data collection

The study cohort consisted of 2,334 patients with AMI from the MIMIC-IV, who were identified based on the Ninth (ICD-9) and Tenth (ICD-10) Revision of International Classification of Diseases.

**Fig. 1:**
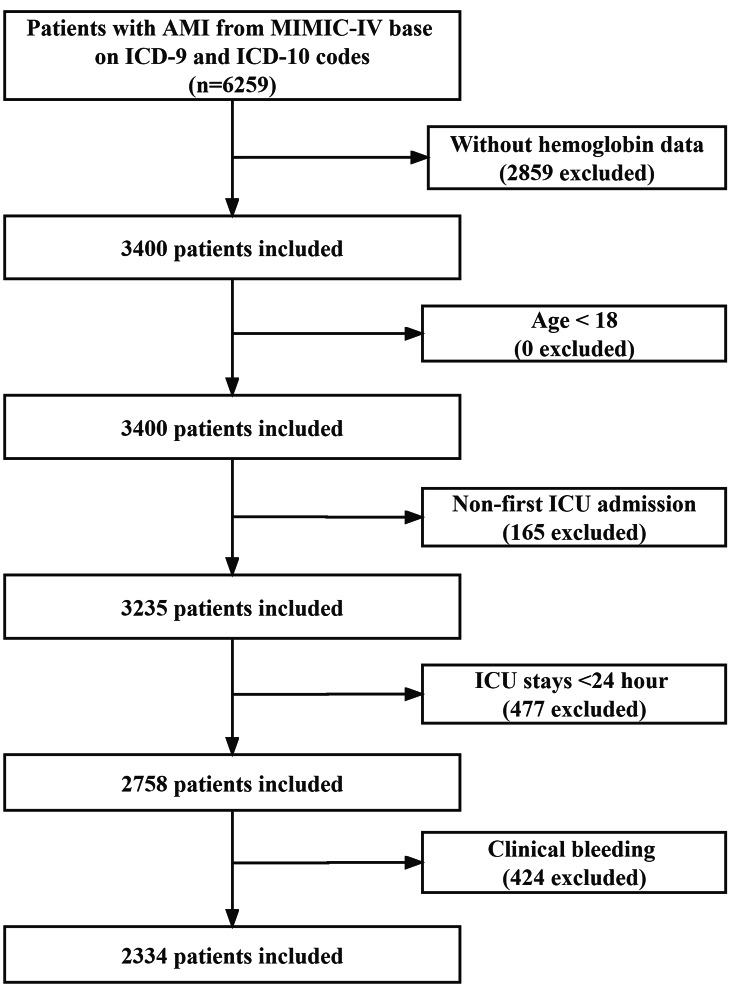
Flowchart of subject screening

Patients with one of the following conditions were excluded: (1) lacked hemoglobin data; (2) less than 18 years old; (3) patients with repeated ICU admissions (If a patient was admitted to the ICU more than once, we used the date of the first admission); (4) less than 24 h of ICU stay; (5) patients diagnosed with clinical bleeding (e.g., gastrointestinal bleeding). The detailed inclusion of our enrolled patients is shown in Fig. 1.

Data on demographics, comorbidities, vital signs, laboratory indicators, invasive treatment, drug use, ICU length of stay (ICU LOS), time of death, and several previously validated risk factors from the Global Registry of Acute Coronary Events (GRACE) risk score [22] were collected within 24 h of initial admission using PostgreSQL (version 14).

1. Demographics included age, gender, weight and ethnicity.
2. Validated risk factors included previous myocardial infarction (Previous MI), ST-segment elevation at presentation (STEMI), non-ST-segment elevation at presentation (NSTEMI), cardiac arrest, Troponin T (TnT) rise and Killip class.
3. Comorbidities included diabetes, hypertension, coronary artery disease (CAD), congestive heart failure (CHF), chronic kidney disease (CKD) and chronic obstructive pulmonary disease (COPD).
4. Vital signs included respiratory rate (RR), heart rate (HR), temperature, systolic blood pressure (SBP), diastolic blood pressure (DBP), mean blood pressure (MBP) and saturation of percutaneous oxygen (SpO2).
5. Laboratory indicators included white blood cell (WBC), red blood cell (RBC), hematocrit, baseline hemoglobin values at admission (baseline hemoglobin), platelet counts (PLT), serum calcium, chloride, potassium, sodium, bicarbonate, anion gap, creatinine, blood urea nitrogen (BUN), international normalized ratio (INR), prothrombin time (PT), activated partial thromboplastin time (APTT), glucose and Troponin T.
6. Scoring systems included the Sequential Organ Failure Assessment score (SOFA), the Oxford Acute Severity of Illness Score (OASIS), the Acute Physiology Score III (APS III) and the Simplified Acute Physiology Score II (SAPS II).
7. Information about treatment consisted of percutaneous coronary intervention (PCI) and coronary bypass artery grafting (CABG).
8. Drug use included antiplatelet agents [Aspirin, P2Y12 inhibitors (Clopidogrel, Prasugrel, Ticagrelor, Cangrelor), and platelet glycoprotein IIb/IIIa receptor inhibitor], anticoagulant agents (Unfractionated heparin, Warfarin, Enoxaparin and Bivalirudin), Anti remodeling agents (ACE inhibitors, Angiotensin II receptor blockers), Beta-blockers and statins.
9. ICU LOS, 28, 60, 90 and 180-day all-cause mortality are also included.

### Outcomes

The primary outcome was 180-day all-cause mortality (since the ICU admission). Mortality at 28 days was also assessed as a secondary outcome.

### Definitions

Baseline hemoglobin was defined as the initial hemoglobin measured at admission, while the nadir value was the lowest hemoglobin level during hospitalization. Patients were grouped according to the degree of hemoglobin drop, which was determined by the positive difference between admission and nadir value. The categorization was as follows: no hemoglobin drop, minimal hemoglobin drop (< 3 g/dl), minor hemoglobin drop (≥ 3 g/dl & < 5 g/dl) and major hemoglobin drop (≥ 5 g/dl).

### Statistical analysis

Continuous variables were shown as mean ± standard deviation (SD) or median (25, 75 percentiles) and analyzed using one-way ANOVA (for normally distributed data) or Kruskal–Wallis H-test (for non-normally distributed data). Categorical variables were presented as frequency (percentage) and tested by Chi-square or Fisher’s exact tests. Missing data were assumed to be randomly missing and completed using multiple imputations.

Patients without hemoglobin drop served as the reference group. Survival distributions among patients with different levels of hemoglobin reduction were compared using the Kaplan-Meier method and log-rank test, and presented as cumulative incidence. The restricted cubic spline (RCS) was utilized to model the nonlinear relationship between mortality and hemoglobin drop as a continuous variable. Proportional hazards (PH) assumption was checked using statistical and graphical tests based on the scaled Schoenfeld residuals.

The variables that violated the PH assumption were defined as time-dependent covariates and then taken as candidates in the time-dependent Cox proportional hazard regression models: a crude model (unadjusted), model 1 (adjusted for demographics, vital signs, several laboratory indicators, PCI and baseline hemoglobin) and model 2 (adjusted for demographics, vital signs, several laboratory indicators, baseline hemoglobin and other potential confounding factors in GRACE) to assess the association of in-hospital hemoglobin drop (as a continuous and a categorical variable respectively) with mortality. Step function was created for each time-dependent covariate to segment survival time and different models were established in different periods. Subgroup analysis of variables was reported by forest plot. All statistical analyses were conducted using STATA (version 16.0) and R software (version 4.1.0). An alpha level of 0.05 was set for statistical significance.

## Results

### Demographic data and baseline characteristics

A total of 2,334 ICU-admitted AMI patients without overt bleeding who met our inclusion and exclusion criteria were included. The baseline characteristics are reported in Table 1. 2,063 patients (88.39%) had hemoglobin reduction with a median value of 1.4 (interquartile range, 0.6–2.5) g/dl. The remaining 271 patients had no hemoglobin reduction.

**Table 1:** Baseline characteristics

	Hemoglobin Drop				
	No Hemoglobin Drop (n = 271)	Minimal (< 3 g/dl) (n = 1661)	Minor (< 3 g/dl&< 5 g/dl) (n = 284)	Major (≥ 5 g/dl) (n = 118)	*P* value
Age, years	70.0 (61.5, 78.0)	68.0 (59.0, 78.0)	67.5 (58.0, 77.0)	67.0 (56.0, 73.0)	0.011
Female, n (%)	90 (33.2)	597 (35.9)	94 (33.1)	31 (26.3)	0.145
Weight, kg	79.0 (68.2, 92.6)	81.6 (69.0, 95.0)	78.0 (68.0, 92.4)	84.5 (73.6, 93.7)	0.075
Ethnicity, n (%)					0.046
White	160 (59.0)	1042 (62.7)	149 (52.5)	72 (61.0)	
Black	17 (6.3)	121 (7.28)	23 (8.1)	4 (3.4)	
Asian	7 (2.6)	35 (2.1)	7 (2.5)	1 (0.9)	
Other	87 (32.1)	463 (27.9)	105 (37.0)	41 (34.8)	
Previous MI	33 (12.2)	181 (10.9)	20 (7.0)	14 (11.0)	0.183
STEMI	28 (10.3)	258 (15.5)	68 (23.9)	40 (33.9)	< 0.001
NSTEMI	179 (66.1)	916 (55.2)	144 (50.7)	42 (35.6)	< 0.001
Troponin T rise	231 (85.2)	1493 (89.9)	264 (93.0)	110 (93.2)	0.013
Cardiac arrest	10 (3.7)	84 (5.1)	31 (10.9)	20 (17.0)	< 0.001
Killip class					< 0.001
I	164 (60.5)	869 (52.3)	129 (45.4)	50 (42.4)	
II	101 (37.3)	692 (41.7)	129 (45.4)	51 (43.2)	
III	2 (0.7)	15 (0.9)	2 (0.7)	4 (3.4)	
IV	4 (1.5)	85 (5.1)	24 (8.5)	13 (11.0)	
*Comorbidities, n (%)*					
Diabetes	76 (28.0)	428 (25.8)	74 (26.1)	29 (24.6)	0.859
Hypertension	97 (35.8)	492 (29.6)	82 (28.9)	35 (29.7)	0.209
CAD	188 (69.4)	1081 (65.1)	185 (65.1)	87 (73.7)	0.155
CHF	79 (29.2)	592 (35.6)	125 (44.0)	52 (44.1)	0.001
CKD	54 (19.9)	277 (16.7)	50 (17.6)	19 (16.1)	0.601
COPD	14 (5.2)	147 (8.9)	29 (10.2)	8 (6.8)	0.129
*Vital signs*					
RR, bpm	18.6 (16.7, 20.7)	18.8 (16.9, 21.2)	19.6 (17.4, 22.3)	20.5 (18.1, 22.9)	< 0.001
h, bpm	80.8 (73.3, 87.6)	80.6 (72.2, 89.5)	83.1 (71.4, 93.0)	81.8 (71.2, 93.2)	0.261
Temperature, °C	36.7 (36.5, 36.9)	36.8 (36.6, 37.0)	36.8 (36.6, 37.1)	36.9 (36.6, 37.2)	< 0.001
SBP, mmHg	112.7 (106.6, 119.4)	112.5 (105.0, 121.3)	109.3 (104.2, 119.2)	111.6 (102.8, 122.2)	0.036
DBP, mmHg	58.4 (52.8, 67.4)	61.0 (55.0, 68.5)	62.0 (56.2, 69.4)	65.6 (59.6, 71.4)	< 0.001
MBP, mmHg	74.4 (70.1, 80.2)	75.9 (70.2, 82.2)	77.3 (72.3, 83.9)	80.9 (74.9, 87.6)	< 0.001
SpO2, %	97.3 (96.0, 98.3)	97.0 (95.8, 98.2)	97.1 (95.8, 98.4)	97.0 (95.3, 98.4)	0.208
*Laboratory Results*					
WBC, ×10^9^/L	14.20 (10.8, 17.8)	13.6 (10.4, 18.3)	15.5 (11.6, 20.9)	17.2 (12.4, 22.4)	< 0.001
RBC, ×10^9^/L	2.9 (2.5, 3.8)	3.5 (3.0, 4.1)	4.0 (3.5, 4.5)	4.5 (3.9, 4.9)	< 0.001
Hematocrit, %	33.8 (30.1, 39.5)	35.20 (31.0, 39.8)	38.80 (34.6, 42.6)	42.8 (39.5, 45.9)	< 0.001
Baseline hemoglobin, g/dl	8.6 (7.4, 11.5)	10.4 (8.9, 12.2)	12.1 (10.9, 13.5)	13.5 (12.5, 14.9)	< 0.001
PLT, ×10^9^/L	211.0 (163.0, 270.0)	198.0 (152.0, 251.5)	211.0 (164.0, 271.0)	214.0 (161.8, 271.3)	0.008
Calcium, mg/dl	8.7 (8.2, 9.1)	8.7 (8.2, 9.2)	8.7 (8.2, 9.1)	8.7 (8.3, 9.1)	0.597
Chloride, mEq/L	105.0 (102.0, 108.0)	106.0 (103.0, 109.0)	105.0 (102.0, 108.0)	106.0 (103.0, 109.0)	< 0.001
Potassium, mmol/L	4.60 (4.2, 5.0)	4.60 (4.3, 5.0)	4.60 (4.2, 5.0)	4.60 (4.2, 5.2)	0.233
Sodium, mmol/L	139.0 (137.0, 141.0)	139.0 (137.0, 141.0)	139.0 (137.0, 141.0)	140.0 (137.0, 142.0)	0.009
Bicarbonate, mEq/L	24.0 (22.0, 26.0)	24.0 (22.0, 26.0)	24.0 (22.0, 26.0)	23.0 (21.0, 26.0)	< 0.001
Anion gap, mEq/L	17.0 (14.0, 20.0)	16.0 (13.0, 19.0)	17.0 (14.00, 20.0)	18.0 (15.0, 21.0)	< 0.001
Creatinine, mg/dl	1.2 (0.9, 1.8)	1.10 (0.9, 1.7)	1.2 (0.9, 1.8)	1.3 (1.0, 1.9)	< 0.001
BUN, mg/dl	23.0 (16.0, 37.0)	21.0 (16.0, 36.5)	22.0 (16.0, 37.0)	25.0 (18.0, 39.0)	0.004
INR	1.3 (1.2, 1.5)	1.4 (1.2, 1.6)	1.3 (1.2, 1.5)	1.30 (1.2, 1.5)	0.045
PT, s	14.4 (12.7, 16.5)	15.0 (12.8, 17.1)	14.4 (12.6, 16.3)	14.2 (12.7, 16.3)	0.052
APTT, s	45.3 (31.1, 79.5)	37.6 (29.7, 61.0)	45.3 (30.8, 74.9)	60.2 (37.3, 109.2)	< 0.001
Glucose, mg/dl	135.6 (119.3, 168.4)	128.0 (117.4, 147.4)	135.7 (119.0, 166.8)	141.2 (122.0, 178.6)	< 0.001
Troponin T, ng/ml	1.6 (0.4, 4.9)	1.4 (0.3, 3.9)	1.6 (0.4, 4.9)	1.3 (0.5, 4.7)	< 0.001
*Score System*					
SOFA	5 (3, 8)	5 (2, 8)	7 (5, 10)	9 (6, 13)	< 0.001
OASIS	31 (24, 37)	31 (25, 38)	36 (29, 43)	35 (28, 42)	< 0.001
APSIII	38 (28, 53)	41 (30, 57)	50 (36, 74)	54 (38, 84)	< 0.001
SAPSII	37 (29, 45)	35 (28, 45)	40 (32, 49)	40 (31, 50)	< 0.001
*Treatment*					
PCI	40 (14.8)	331 (19.9)	30 (10.6)	13 (11.0)	< 0.001
CAGB	17 (6.3)	65 (3.9)	20 (7.0)	17 (14.4)	< 0.001
*Medications*					
Antiplatelet agents	254 (93.7)	1571 (94.6)	267 (94.0)	112 (94.9)	0.925
Anticoagulation agents	243 (89.7)	1548 (93.2)	273 (96.1)	118 (100.0)	< 0.001
Antiremodeling agents	112 (41.3)	848 (51.1)	140 (49.3)	47 (39.8)	0.004
Beta-blockers	234 (86.4)	1415 (85.2)	231 (81.3)	99 (83.9)	0.332
Statins	245 (90.4)	1520 (91.5)	254 (89.4)	101 (85.6)	0.137

Data are presented as median (interquartile range), or number of patients (%)

MI, myocardial infarction; STEMI, ST-segment elevation myocardial infarction; NSTEMI, non-ST-segment elevation myocardial infarction; CAD, coronary artery disease; CHF, congestive heart failure; CKD, chronic kidney disease; COPD, chronic obstructive pulmonary disease; RR, respiratory rate; HR, heart rate; SBP, systolic blood pressure; DBP, diastolic blood pressure; MBP, mean blood pressure; SpO2, percutaneous oxygen saturation; WBC, white blood cell; RBC, red blood cell; PLT, platelet; BUN, blood urea nitrogen; INR, international normalized ratio; PT, prothrombin time; APTT, activated partial thromboplastin time; SOFA, sequential organ failure assessment; OASIS, oxford acute severity of illness score; APSIII, acute physiology score III; SAPSII, Simplified Acute Physiology Score II; PCI, percutaneous transluminal coronary intervention; CABG, coronary artery bypass grafting

The population was divided into four groups based on hemoglobin drop severity: No hemoglobin drop group (N = 271, 11.61%); Minimal hemoglobin drop group (< 3 g/dl, N = 1661, 71.17%); Minor hemoglobin drop group (≥ 3 g/dl&< 5 g/dl, N = 284, 12.17%); Major hemoglobin drop group (≥ 5 g/dl, N = 118, 5.06%).

The patients were aged 68 (interquartile range, 59–77) years and comprised 812 (34.79%) females. Baseline characteristics varied considerably among the four groups. In general, increasing hemoglobin reduction is associated with more comorbidities, higher scoring systems, and higher proportions of patients with STEMI, cardiac arrest, Killip Class and TnT rise. Regarding treatment, the prevalence of PCI, CAGB, anticoagulant agents and anti-remodeling agents progressively increased with the aggravation of hemoglobin reduction. Notably, younger patients were associated with higher hemoglobin reduction. No significant differences were observed in gender, weight, previous MI, Spo2, hypertension, diabetes, CKD, CAD, COPD, calcium, INR, PT, or potassium.

### Clinical outcomes

Table 2 displays the unadjusted clinical outcomes comparing the four groups. A total of 355 patients (15.21%) died during 180 days. Patients with minor or major hemoglobin drop (≥ 3 g/dl) had significantly higher 28-day, 60-day, 90-day, and 180-day all-cause mortality. Furthermore, the severity of hemoglobin reduction was associated with a longer ICU LOS.

**Table 2:** Outcomes

Outcomes	All patience (n = 2334)	No Hemoglobin Drop (n = 271)	Minimal (< 3 g/dL) (n = 1661)	Minor (≥ 3 g/dL&< 5 g/dL) (n = 284)	Major (≥ 5 g/dL) (n = 118)	P value
ICU mortality, n (%)	226 (9.7)	25 (9.2)	142 (8.6)	35 (12.3)	24 (20.3)	< 0.001
ICU LOS (days)	2.4 (1.5, 4.3)	1.7 (1.3, 3.0)	2.2 (1.4, 4.0)	3.9 (2.4, 6.9)	5.1 (2.8, 11.1)	< 0.001
Hospital mortality, n (%)	304 (13.0)	34 (12.6)	192 (11.6)	51 (18.0)	27 (22.9)	< 0.001
28-day mortality, n (%)	301 (12.9)	33 (12.2)	193 (11.6)	51 (18.0)	24 (20.3)	0.002
60-day mortality, n (%)	328 (14.1)	36 (13.3)	209 (12.6)	56 (19.7)	27 (22.9)	< 0.001
90-day mortality, n (%)	341 (14.6)	38 (14.0)	217 (13.1)	58 (20.4)	28 (23.7)	< 0.001
180-day mortality, n (%)	355 (15.2)	40 (14.8)	224 (13.5)	63 (22.2)	28 (23.7)	< 0.001

ICU LOS, ICU length of stay

### The association between hemoglobin drop and clinical outcome

**Fig. 2:**
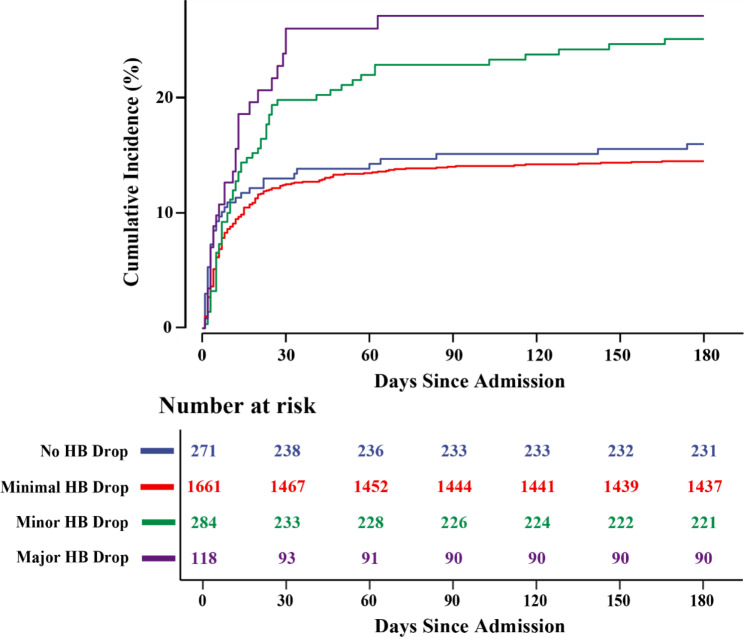
Cumulative incidence of 180-day all-cause mortality stratified by hemoglobin (HB) drop

Figure 2 shows that non-overt bleeding AMI patients with a drop in hemoglobin levels, whether minor or major, have a higher cumulative incidence of 180-day all-cause mortality than those with minimal or no hemoglobin drop (log-rank test: P < 0.001).

After stratification of the period (cut by 6 days) for the univariate and multivariate time-dependent Cox proportional hazard regression models, variables and the whole model satisfied the PH assumption (*P* > 0.05). We found an independent association between hemoglobin drop and all-cause mortality in non-overt bleeding ICU patients with AMI. In Table 3, we displayed the unadjusted and adjusted models for the second period.

**Table 3:** Univariate and multivariate time-dependent Cox regression analysis for 28-day and 180-day all-cause mortality

	Cohort-Univariate		Model 1-Multivariate		Model 2-Multivariate	
	Crude HR (95% CI)	*P* value	Adjusted HR (95% CI)	*P* value	Adjusted HR (95% CI)	*P* value
28-day Mortality						
No hemoglobin drop	Ref.	-	Ref.	-	Ref.	-
Minimal hemoglobin drop	2.38 (1.02–5.55)	0.044	3.44 (1.45–8.18)	0.005	3.44 (1.45–8.20)	0.005
Minor hemoglobin drop	5.09 (1.92–13.48)	0.001	12.90 (4.51–36.92)	< 0.001	14.31 (4.99–41.06)	< 0.001
Major hemoglobin drop	3.30 (1.07–10.20)	0.038	15.81 (4.42–56.56)	< 0.001	16.15 (4.47–58.32)	< 0.001
Continuous hemoglobin drop	1.24 (1.14–1.34)	< 0.001	1.60 (1.38–1.86)	< 0.001	1.58 (1.37–1.84)	< 0.001
180-day Mortality						
No hemoglobin drop	Ref.	-	Ref.	-	Ref.	-
Minimal hemoglobin drop	1.75 (0.88–3.49)	0.113	2.75 (1.35–5.60)	0.005	2.75 (1.35–5.60)	0.005
Minor hemoglobin drop	3.83 (1.67–8.79)	0.002	11.79 (4.76–29.10)	< 0.001	12.68 (5.13–31.33)	< 0.001
Major hemoglobin drop	2.40 (0.89–6.41)	0.082	13.90 (4.52–42.73)	< 0.001	13.87 (4.50-42.76)	< 0.001
Continuous hemoglobin drop	1.22 (1.14–1.30)	< 0.001	1.61 (1.40–1.85)	< 0.001	1.34 (1.22–1.47)	< 0.001

CI, confidence interval; HR, hazard ratio

After adjusting for demographics, vital signs, several laboratory indicators, PCI and baseline hemoglobin in Model 1, and for additional confounders (previous MI, comorbidities, TnT rise, Killip Class and cardiac arrest) in Model 2, the excess risks of the minimal, minor and major hemoglobin drop relative to no hemoglobin group were significant for the 180-day mortality. The hazard of 28-day mortality for minimal, minor and major hemoglobin drop was directionally similar but had wider CIs in Model 1 and Model 2. Modeling the hemoglobin drop as a continuous variable revealed a consistent increase in the hazard of 180-day mortality in all models.

### The nonlinear relationship between hemoglobin drop and 180-day all-cause mortality

**Fig. 3:**
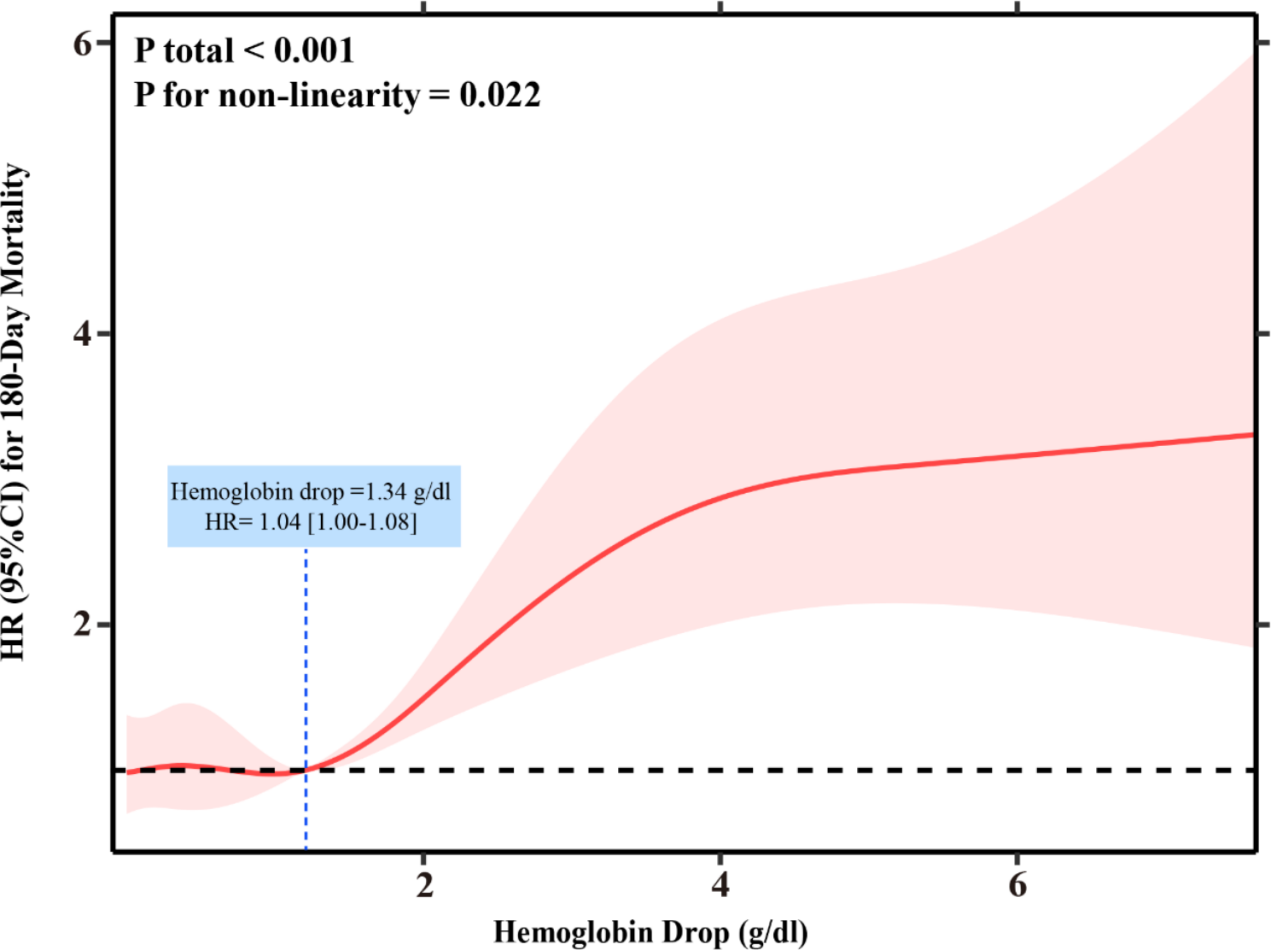
The nonlinear relationship between 180-day All-Cause Mortality and hemoglobin drop after adjusting baseline value

After adjusting baseline hemoglobin levels, we observed a nonlinear relationship between hemoglobin drop and 180-day all-cause mortality in non-overt bleeding ICU patients with AMI (*P* for interaction = 0.137), with the risk of mortality remaining constant until a hemoglobin drop of 1.34 g/dl, beyond which it began to increase (P for non-linearity < 0.05). This relationship is visualized in Fig. 3 using restricted cubic splines.

### Subgroup analysis

**Fig. 4:**
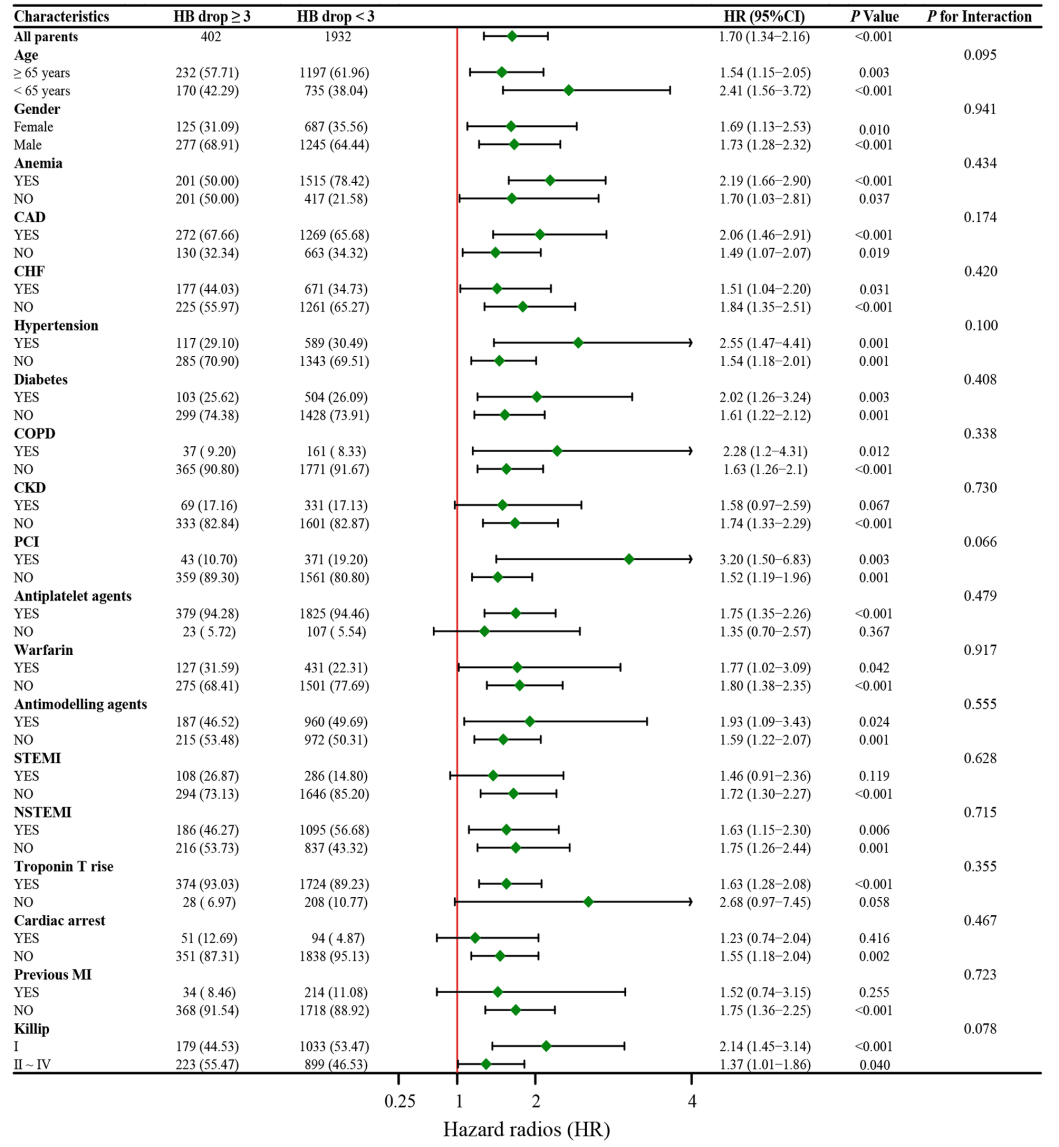
Association between hemoglobin drop ≥ 3 g/dl and 180-day all-cause mortality of non-overt bleeding patients with AMI in different subgroups CAD, coronary artery disease; CHF, congestive heart failure; COPD, chronic obstructive pulmonary disease; CKD, chronic kidney disease; PCI, percutaneous transluminal coronary intervention; STEMI, ST-segment elevation myocardial infarction; NSTEMI, non-ST-segment elevation myocardial infarction; MI, myocardial infarction

Figure 4 shows that the relationship between hemoglobin drop and 180-day all-cause mortality was consistent across all subgroups, with no significant interaction with any baseline factors. However, AMI patients with hemoglobin drop ≥ 3 g/dl had a higher risk of 180-day mortality than those with minimal or no hemoglobin drop in most subgroups, except for patients with CKD, STEMI, non-Troponin T rise, cardiac arrest, and previous MI, where the difference was not statistically significant.

## Discussion

Our study found a potential association between hemoglobin drop and all-cause mortalities in non-overt bleeding ICU patients with AMI. Key findings include:

1. hemoglobin drop is common during hospitalization in non-overt bleeding ICU-admitted patients with AMI;
2. a hemoglobin drop of ≥ 3 g/dl during hospitalization was independently associated with 180-day all-cause mortalities regardless of invasive management. The adjusted risk of 180-day all-cause mortality increased by 34% for each 1 g/dl hemoglobin drop;
3. after adjusting for baseline hemoglobin, there was a robust nonlinear relationship between in-hospital hemoglobin drop and increased 180-day mortality, with the lowest value associated with increased mortality being 1.34 g/dl.

### The clinical value of hemoglobin level in patients with AMI

The prevalence of anemia has been associated with notably increased mortality and poor prognosis in multiple diseases, including ACS and heart failure [23–25]. Prior researches has primarily examined the clinical significance of hemoglobin levels at a specific time. However, decreasing baseline hemoglobin levels, even within the normal range, have been correlated with higher long-term risk for bleeding events, ischemic stroke, and mortality after PCI [23, 26, 27]. This study expands on previous research by investigating the clinical impact of in-hospital hemoglobin drop in non-overt bleeding AMI patients.

### Hemoglobin decline in bleeding definitions of AMI patients

Revascularization strategies and evidence-based antithrombotic therapies have significantly improved the prognosis of patients with AMI [3]. However, bleeding complications remain a significant concern and are associated with unfavorable outcomes, including recurrent MI and higher mortality [8, 28]. In clinical practice, the proportion of patients with overt bleeding events is low. Numerous potential bleeding events may remain undetected.

As a simple, affordable and easily implemented biochemical indicator, various cut-off values of hemoglobin drop have been used to evaluate bleeding in patients with AMI. However, there is no consensus on the cut-off values for defining bleeding severity [13–17, 29]. Current definitions are based mainly on overt bleeding events, and there is limited evidence on the significance of hemoglobin drop in non-overt bleeding AMI patients.

### The consequences of hemoglobin drop without overt bleeding and clinical implications

Hemoglobin level variations are common in non-overt bleeding patients hospitalized for ACS, particularly those who undergo PCI. Leonardi et al. reported that a hemoglobin drop ≥ 3 g/dl was independently associated with 1-year mortality in ACS patients without overt bleeding events [19]. Ndrepepa et al. showed that a hemoglobin drop as small as 1.13 g/dl in non-overt bleeding patients was associated with an increased risk of 1-year mortality [30]. Salisbury et al. showed that moderate-severe hospital-acquired anemia was associated with higher mortality and worse clinical condition [31]. The causes of hemoglobin decline remain unclear, but multiple mechanisms have been studied, including stress-induced hemodilution [32], fluid overload resulting from heart failure [33, 34], increased inflammatory status [35], blood loss from the procedure or frequent blood draws [36], rapid fluid infusion [37], and erythropoiesis suppression due to cytokines produced by myocardial necrosis [11]. Subclinical bleeding caused by aggressive anticoagulation has been suggested as a possible reason [38]. Previous study observed that physicians may overreact on in-hospital hemoglobin drop by withdrawing antiplatelet agents or using excessive packed red blood cells transfusion, as those may worsen the patient’s prognosis [39, 40]. The findings highlight the need to exercise caution about the in-hospital hemoglobin drop in critically ill patients, to consider the underlying reasons for the decline, and to treat them symptomatically.

### Prognostic value of hemoglobin drop in ICU-admitted patients with AMI

Patients with AMI admitted to the ICU often have a worse condition, a lower possibility of invasive intervention, and are more likely to be exposed to multiple drugs that promote bleeding. To our knowledge, only few studies have evaluated the prognostic value of hemoglobin decline in non-overt bleeding ICU patients hospitalized for AMI, potentially due to sample size restrictions and inadequate hemoglobin assessments. Previsdomini et al. suggested that hemoglobin decline may occur in ACS patients without any sign during ICU stay, occurring in roughly 88% of nonbleeders with an average of 1.27 ± 1.00 g/dl decrease, but the study just concerned 103 white patients [41]. A similar conclusion was reached by Mare´chaux et al. by analyzing 591 ICU-admitted patients with ACS, where hemoglobin drop ≥ 0.9 g/dl (32% of the population) was associated with a higher death risk [24]. Our study investigated the clinical significance of in-hospital hemoglobin drop in 2,334 ICU-admitted AMI patients after excluding those who suffered overt bleeding events. We found a robust correlation between the hemoglobin drop during hospitalization and all-cause mortality. Minor to major degree (≥ 3 g/dl) of hemoglobin drop was significantly associated with 180-day mortality compared with no hemoglobin drop.

It has been observed that a low hemoglobin level at baseline was an independent predictor of major bleeding, long-term ischemic, death and MI, even in ACS patients without anemia [23, 26, 42]. Most strikingly, patients with hemoglobin drop have higher baseline hemoglobin than those without hemoglobin drop in our study. Obviously, a hemoglobin drop is more likely to occur with a higher baseline value. After adjusting baseline hemoglobin as a covariate purposefully in our study, the degree of hemoglobin drop was still associated with higher 180-day mortality. Corroborating the prior studies of patients undergoing PCI, the effect of minor to major hemoglobin drop (≥ 3 g/dl) on the clinical outcome was prominent regardless of baseline anemia [43].

Risk stratification is a crucial factor in the decision of revascularization or medical therapy. An observational, multicenter cohort study of 24,189 patients with ACS showed that a significant proportion of high-risk patients might not be technically suitable for revascularization. In addition, the comorbidities such as diabetes and chronic heart failure may lead to unacceptable periprocedural risk [44]. In clinical, ACS patients who received revascularization were paid more attention than those who did not. And the prescription rate and compliance with antiplatelet therapy were also significantly higher. The EPICOR-Asia study confirmed that NSTE-ACS patients without revascularization had significantly higher all-cause mortality within two years than patients with revascularization (8.7% vs. 3.0%; *P* < 0.001) [45]. Compared with revascularized ACS patients, non-revascularized patients have a higher risk of adverse outcomes and poor prognosis, so special attention should be paid to these patients. In our study, only 17.74% of ICU patients received PCI. Subgroup analysis revealed a significant association between hemoglobin drop and 180-day all-cause death in patients with or without PCI. It stands to reason that hemoglobin drop maintained its predictive power in ICU patients without PCI.

### Limitation

Several limitations should be considered for this study. Firstly, as a single-center retrospective observational study, selection bias was inevitable. Patients without baseline hemoglobin values at admission were excluded, which also may lead to selection bias. Secondly, patients’ information was identified using ICD-9, ICD-10 and unique ID from the MIMIC-IV database rather than clinical diagnostic criteria, so some information was not specific. Thirdly, since it is difficult to obtain accurate and detailed information from the database, factors such as blood transfusions, blood diseases and malignant tumors that may affect the level of hemoglobin drop were not excluded as exclusion criteria, which may result in some misclassification. Fourthly, the results of the study could have still been affected by residual confounding despite extensive statistical adjustments for potential confounders. Finally, hemoglobin values were unavailable after hospital discharge, consequently, we cannot analyze the prognostic impact of persistent hemoglobin drop after discharge. The optimal hemoglobin drop cut-off value for various populations should be determined with further external validation based on large multicenter studies.

## Conclusions

In non-overt bleeding ICU-admitted patients with AMI, in-hospital hemoglobin drop ≥ 3 g/dl was independently associated with a higher risk of all-cause mortality at 180-day regardless of baseline hemoglobin and invasive treatment. To improve the prognosis of these high-risk ICU patients, more attention should be paid to the reasons for hemoglobin drop and particular therapeutic strategies should be researched.

## Electronic supplementary material

Below is the link to the electronic supplementary material.

Additional File: TRIPOD Checklist: Prediction Model Development

## Data Availability

The original data of our study are available from the corresponding author upon reasonable request.
